# Introduction. RSV and RAD: possibilities for prevention? The link between respiratory syncytial virus and reactive airway disease

**DOI:** 10.1186/rr182

**Published:** 2002-06-24

**Authors:** Xavier Carbonell-Estrany, Jan LL Kimpen

**Affiliations:** 1 Servei de Neonatologia, Hospital Clinic, Institut Clinic de Ginecologia, Obstetrica i Neonatologia, Barcelona, Spain; 2 Wilhelmina Children's Hospital, University Medical Center Utrecht, Utrecht, The Netherlands

## 

Respiratory syncytial virus (RSV) is a common cause of lower respiratory tract infections in infants and small children worldwide. There is no cure for RSV disease, but immunoprophylaxis using humanized monoclonal antibody has proven to be safe and efficacious in preventing disease. Extensive research supports the hypothesis that RSV bronchiolitis in infancy results in increased risk for development of wheezing, asthma, and possibly atopy throughout childhood. In spite of this, the pathophysiologic mechanisms that are responsible for this association have not been fully elucidated.

We recently co-chaired an international panel of physicians that met to discuss the link between RSV and reactive airway disease (RAD). Research results in the clinical arena and current opinion on RSV prophylaxis and the RSV-RAD association are presented in this special supplement to *Respiratory Research*.

Professor Rosalind Smyth from the University of Liverpool, UK, presented evidence supporting the argument that asthma is a major pediatric health issue. The incidence of asthma has increased considerably over the past three decades in the UK, although since the early 1990s the incidence of new episodes has declined. This overall increase has been observed worldwide, but there is broad variation between countries. Environmental factors, genetic factors, the hygiene hypothesis, and lifestyle differences may play roles in causing asthma, but currently there is no unifying explanation for these trends. One hypothesis for the etiology of asthma suggests that asthmatic children have normal lung function at birth, but inflammatory and immunologic events in the airways, such as those seen in RSV bronchiolitis during infancy, contribute to persistence of wheeze.

Dr Nele Sigurs of Borås Central Hospital, Sweden, presented clinical perspectives on the association between RSV and RAD. She reviewed the eight studies to date that have compared children with and without RSV bronchiolitis, and all have found an increased incidence of bronchial obstructive symptoms in the ex-bronchiolitics. There were no significant differences in family history of atopy or asthma in any of those studies. An increased risk for allergic sensitization was observed in three of the studies. It remains unclear whether RSV bronchiolitis alone is responsible for these symptoms or whether other risk factors contribute. Further studies are needed to investigate the effect of RSV interventional agents in order to resolve this issue.

Professor Peter Openshaw of the Imperial College of Science, Technology and Medicine in London addressed the question of whether RSV is a cause or consequence of airway pathology. He reviewed the various inflammatory and cytokine responses associated with RSV infection and showed that data from animal studies support the possibility that wheezing in childhood results from viral bronchiolitis in infancy. The timing of severe disease appears to be critical for the development of pulmonary sequelae, and preventing or delaying RSV infection could reduce the number of children who suffer from recurrent wheeze during childhood. He concurred that prospective interventional studies are needed.

Professor Giovanni Piedimonte of the University of Miami, Florida, USA, proposed pathophysiologic mechanisms for the RSV-RAD link, based on his studies in rats. He has observed upregulation of receptors for substance P, a proinflammatory neuropeptide, in T-lymphocyte subpopulations within the bronchial-associated lymphoid tissue of RSV-infected rats. He postulates that this mechanism establishes important neuroimmune interactions that result in long-term dysregulation, predisposing to airway inflammation and hyperreactivity. More importantly, these changes were avoided if rats were administered palivizumab, a monoclonal antibody to the fusion protein of RSV, before or during the early phase of infection. Therefore, results in animal studies suggest that prevention of RSV lower respiratory tract infection with anti-RSV antibody protects against respiratory sequelae associated with this virus.

Professor Eric Simoes of The Children's Hospital in Denver, Colorado, USA, reviewed the global experience of immunoprophylaxis for RSV. He presented a combined analysis of several studies that have reported hospitalization rates for RSV in children who had received palivizumab and in children who did not receive prophylaxis. The children were divided into three subgroups: those with chronic lung disease (CLD) and/or bronchopulmonary dysplasia (BPD); premature infants born at gestational age 29–32 weeks without CLD/BPD; and premature infants born at gestational age 32–35 weeks without CLD/BPD. The hospitalization rates were significantly lower in the children who had received palivizumab for all three groups. He also reviewed several studies conducted in Europe and North America that continue to support the safety and efficacy of palivizumab.

Dr Lone Graff Stensballe of the Statens Serum Institut in Copenhagen, Denmark, presented the design of a study she has initiated in Denmark to investigate prospectively the incidence of RSV infection in Denmark and factors that predispose to or protect against serious RSV disease, and to examine the association between severe RSV infection and RAD. Several population-based registers will be used to examine the influence of various biologic, social, and environmental factors on hospitalization for RSV disease.

Professor Jan Kimpen of the University Medical Center Utrecht, The Netherlands, concluded the session with his examination of the impact of prevention and treatment of RSV on development of RAD. His review of published studies revealed that ribavirin does not appear to reduce significantly the long-term respiratory morbidity associated with RSV bronchiolitis. Corticosteriod or bronchodilator therapy may improve outcomes, but only on a short-term basis. Investigations are underway to determine whether immunoprophylaxis reduces the incidence of RAD in children.

## Abbreviations

BPD = bronchopulmonary dysplasia; CLD = chronic lung disease; RAD = reactive airway disease; RSV = respiratory syncytial virus.

